# Enhanced relapse prevention for bipolar disorder – ERP trial. A cluster randomised controlled trial to assess the feasibility of training care coordinators to offer enhanced relapse prevention for bipolar disorder

**DOI:** 10.1186/1471-244X-7-6

**Published:** 2007-02-02

**Authors:** Fiona Lobban, Carol Gamble, Peter Kinderman, Lee Taylor, Claire Chandler, Elizabeth Tyler, Sarah Peters, Eleanor Pontin, William Sellwood, Richard K Morriss

**Affiliations:** 1 School of Psychological Sciences, Faculty of Medical and Human Sciences, University of Manchester, Manchester, UK; 2 Centre for Medical Statistics and Health Evaluation, Faculty of Medicine, University of Liverpool, Liverpool, UK; 3 Division of Clinical Psychology, Faculty of Medicine, University of Liverpool, Liverpool, UK; 4 Forensic Division, Penninecare NHS Trust, Lancashire, UK; 5 School of Community Health Sciences, Faculty of Medicine and Health Sciences, University of Nottingham, UK

## Abstract

**Background:**

Bipolar Disorder (BD) is a common and severe form of mental illness characterised by repeated relapses of mania or depression. Pharmacotherapy is the main treatment currently offered, but this has only limited effectiveness. A recent Cochrane review has reported that adding psycho-social interventions that train people to recognise and manage the early warning signs of their relapses is effective in increasing time to recurrence, improving social functioning and in reducing hospitalisations. However, the review also highlights the difficulties in offering these interventions within standard mental health services due to the need for highly trained therapists and extensive input of time. There is a need to explore the potential for developing Early Warning Sign (EWS) interventions in ways that will enhance dissemination.

**Methods and design:**

This article describes a cluster-randomised trial to assess the feasibility of training care coordinators (CCs) in community mental health teams (CMHTs) to offer Enhanced Relapse Prevention (ERP) to people with Bipolar Disorder. CMHTs in the North West of England are randomised to either receive training in ERP and to offer this to their clients, or to continue to offer treatment as usual (TAU). The main aims of the study are (1) to determine the acceptability of the intervention, training and outcome measures (2) to assess the feasibility of the design as measured by rates of recruitment, retention, attendance and direct feedback from participants (3) to estimate the design effect of clustering for key outcome variables (4) to estimate the effect size of the impact of the intervention on outcome. In this paper we provide a rationale for the study design, briefly outline the ERP intervention, and describe in detail the study protocol.

**Discussion:**

This information will be useful to researchers attempting to carry out similar feasibility assessments of clinical effectiveness trials and in particular cluster randomised controlled trials.

## Background

Bipolar Disorder (BD) is a common and severe form of mental illness characterised by repeated relapses of mania or depression. Recurrence rates are high at around 50% at one year and 70% at four years [1,2]. Pharmacotherapy is the main treatment currently offered, but this has only limited effectiveness [3]. Surveys of patient organisations in the United States (US) and United Kingdom (UK) reveal a strong wish by patients for both self-help and psychological treatments in addition to pharmacotherapy [4,5]. One form of intervention is to teach patients with BD to recognise and manage early warning signs (EWS) of mania and depressive episodes. A recent systematic review of this approach found that 11 RCTs involving 1324 patients show the efficacy of interventions that include this approach [6]. Overall, EWS interventions, in addition to treatment as usual, increased time to recurrence and reduced the percentage of people hospitalised. Despite having no clear impact on depressive or manic inter-episode symptoms, there was some evidence that EWS interventions had a positive impact on levels of functioning.

Most of these studies reviewed involved interventions consisting of extensive hours of therapy with highly trained therapists, clearly restricting their generalisability to current NHS services. The review highlights the need for further research to explore cost-effective ways in which these interventions can be offered within the NHS. Bauer et al (2006) showed that positive outcomes for reducing relapse into mania could be achieved within Veterans Affairs hospitals in the US using a chronic care model in which people are transferred to a specialist team providing group psychoeducation, improved pharmacotherapy guidelines, and coordinated care to enhance communication within services [7,8]. Although demonstrated to be cost-neutral, this intervention required setting up new specialist bipolar disorder teams and the benefits for service users were only apparent by the third year of being in such a service [7]. Although designed to address issues of external validity, generalising from these settings to the UK NHS and probably other health settings is problematic because there is no political will to provide brand new services specifically and only for service users with BD [9]. However, there is political will to adapt existing services to meet the needs of service users with BD more specifically.

The least resource intensive intervention in a UK study was that by Perry et al (1999) in which a median of 9 sessions were offered by a minimally trained research psychologist in addition to treatment as usual (TAU) and demonstrated a fourfold increase in time to next manic episode in people receiving Relapse Prevention (RP) compared to those receiving treatment as usual only, and a 30% reduction in number of manic episodes over 18 months[10]. The intervention did not detect an impact on depressive episodes. This is likely to be due to greater difficulty in identifying EWS of depression, lack of clear coping strategies for these signs, and delay in response to anti-depressant medication.

In this study we have devised an enhanced form of a RP approach, which is enhanced by an increased focus on the identification of EWS for depression, more detailed development of coping strategies available for both depression and mania, and the involvement of a relative/friend to support the intervention where appropriate. The key elements of the ERP intervention are those explicitly recommended by the NICE Guidelines for Bipolar Disorder [9], and include psychoeducation, a detailed analysis of previous episodes, identification of trigger situations and early warning signs, enhancing coping strategies for mood changes, an action plan for responding to early warning signs, and an agreement with clinical services about how they will respond to different stages of relapse. These elements are done separately for mania, depression and mixed episodes. The intervention is used alongside other interventions such as pharmacotherapy.

In addition to the enhancements to the RP approach, this study differs significantly from those described previously (including Perry et al (1999)) in that the ERP intervention will be offered to people with Bipolar Disorder by Care Coordinators (CCs) currently working in Community Mental Health Teams (CMHTs) in the National Health Service (NHS). A CMHT is a multidisciplinary team of health professionals (community mental health nurses, occupational therapists, psychiatric social workers, clinical psychologists and psychiatrists) working out of a team base in the geographical locality the CMHT serves. The CMHT sees service users at the base, other clinic bases or in the home of the service user. All service users with serious mental illness whose needs require more than the support of one professional group would be looked after by a CMHT with one of the health professionals in the team acting as a CC taking the lead role for coordinating the care of the patient but not delivering all aspects of the patient's care [11]. All service users in the CMHT will have as a minimum a CC and a psychiatrist but there may also be other members of the CMHT or other health or social service professionals who would provide specific services to the service user with BD. Hence, this study focuses on issues related to the clinical effectiveness of relapse prevention in a real world setting that uses existing CCs and is able to examine the impact and the barriers that arise in this situation. We are aiming to address the important issue of transferring evidence-based interventions from psychological research settings into NHS practice. Interventions that have been shown to be clinically efficacious under ideal conditions may not be effective in clinical practice [12]. The approach is consistent with the Department of Health remit for CMHTs to offer psychological interventions aimed at preventing relapse in people with severe mental illnesses ("such as schizophrenia and bipolar disorder") and to work with both patients and carers [11].

The approach relies on a quick and reliable response from the CMHTs to changes in patients' symptoms and therefore the intervention must be offered by the team as a whole, rather than by individual CCs. Once trained, these teams would be unable to offer treatment as usual. Thus in order to assess the effectiveness of the intervention, a cluster randomised controlled trial (RCT) is needed in which the CMHT is the unit of randomisation. Effectiveness can be assessed at two levels; firstly, the effectiveness of the training on the ability of the CCs to carry out the key elements of the ERP interventions; secondly the effectiveness of the CC delivered ERP intervention on reducing relapse rates in people with Bipolar Disorder.

A large-scale evaluation of the effectiveness of the ERP intervention is required. However, this is likely to be a very expensive study due to the costs of training staff and the recruitment, assessment and retention of large numbers of staff, service users and relatives. Therefore it is important that the study is theoretically well designed, methodologically feasible, adequately powered to identify any impact the intervention has on outcome, and well executed. To enable this to happen, essential information is required that will be obtained from this study. This includes: an understanding of what current TAU is for people with Bipolar Disorder who are offered a service by CMHTs; acceptability of the ERP intervention, training and supervision packages; effectiveness of training and supervision on Care Coordinator competency and confidence to offer ERP; recruitment and retention rates for CMHTs, individual Care Coordinators, service users with BD and relatives; acceptability, validity and reliability of assessment measures including those modified for the study; identification of major barriers to carrying out the project within the NHS; an estimate of the effect size of the intervention; an estimate of the design effect of clustering. Detailed evaluation of process variables will allow the mechanism of action to be understood so that important inferences can be drawn in terms of the wider implementation of the intervention. This trial is an example of a Phase II exploratory trial in the Medical Research Council (MRC) framework for development and evaluation of RCTs for complex interventions to improve health [13]. Reporting the details of the study design also contributes to a widely recognised need for increased sharing of information from pilot studies of cluster trials to improve methodological developments in this area [14].

The aim of the trial is to assess the feasibility of a large cluster randomised trial to evaluate the effectiveness of training CCs in ERP for people with bipolar disorder.

The aim of this paper is to outline the ERP intervention, provide a rationale for the study design, and describe in detail the study protocol. Future papers will give a detailed description of the intervention and the process of training and will report on the feasibility and effectiveness of the training and the impact of this on relapse rates for people with Bipolar Disorder.

## Methods

### Structure of the trial

The trial is conducted by multidisciplinary researchers based across 3 academic institutions, and CMHT staff working in the NHS. The trial is funded by the Medical Research Council and Merseycare NHS Trust, sponsored by the University of Liverpool, and is supported by an independent Trial Steering Committee consisting of service users, researchers, clinicians and statisticians. Ethical approval through Central Office for Research Ethics Committees (COREC) and Research & Development (R&D) approval at each Trust has been given.

### Design

ERP is a stratified cluster randomised controlled trial with extensive evaluation of the process as well as the outcome of the trial. CMHTs are randomly allocated to receive training and supervision in ERP or to continue to offer Treatment as Usual (TAU). The number of teams in each Trust is identified prior to randomisation. The variable for stratification is NHS Trust. This is to take into account different systems and approaches for working with Bipolar Disorder between Trusts and also any changes in guidelines for working with BD that may appear during the period of study. Teams are allocated numbers, which are then randomised electronically. Service users with BD from teams in both arms are assessed by researchers blind to allocation on a range of outcome measures for up to 48 weeks following the onset of treatment. All incidences of unblinding are recorded.

A cluster design is considered necessary because all members of a CMHT need to be able to understand and implement an individual relapse prevention plan for all service users. Although each service user has a named CC, other members of the team are closely involved in their care through out-of-hours cover, crisis management, co-working etc. Therefore any training aimed at modifying practice should be offered to teams rather than individuals. Data from service users with Bipolar Disorder who are within the same cluster i.e. seen by the same team, are not independent. Other variables, such as team set-up, previous team training, management style, etc. are likely to influence the outcome of the intervention. Failing to account for this clustering effect is may result in under-powered studies [15].

### Sample

#### Sample size

A power calculation is not applicable for this study. The aim of the feasibility study is to collect information that will allow us to estimate the effect size for ERP compared to TAU and to estimate the impact of using a cluster randomised design (intra-class correlation coefficient). This information will allow a meaningful sample size calculation to be made for a large-scale definitive effectiveness trial (Phase III within the MRC complex interventions framework).

#### Inclusion/Exclusion criteria

All CMHTs across 4 North West NHS Trusts are invited to take part in the trial. Specialist teams such as Assertive Outreach Teams (AOTs), Crisis Teams, Home Treatment Teams, and Early Intervention Teams are excluded.

Teams are included in the randomisation if a minimum of 4 CCs gives informed consent to take part in the study. This includes agreeing to attend the training and supervision and to offer the intervention to a minimum of 3 service users each if the team is allocated to ERP. CMHTs working from the same building with extensive clinical cover are entered as one team. This reduces the possibility of any contamination between teams allocated to different arms of the trial.

Staff with a range of backgrounds are invited to take part in the training (nurses, occupational therapists, and social workers), but must be identified Care Coordinators for service users. Support workers are excluded from the study but are invited to sit in on the training where resources allowed.

Service users are invited to take part if they had a lifetime diagnosis of Bipolar I or II, have experienced two or more relapse ever and at least one in the last year or two in the last three years, and are currently not in a major depressive, hypomanic, manic or mixed episode in the last four weeks. Service users are excluded if there is a clear organic cause of their disorder, they have a rapid cycling disorder, they have significant cognitive impairment, they have alcohol/drug misuse as a primary diagnosis, they do not have a working understanding of English language, or they are unable or unwilling to give informed consent.

#### Recruitment and consent

All CMHTs in the participating NHS Trusts are given a verbal presentation about the study and written information sheets. A minimum of 4 CCs from each team are asked to give written consent to invite service users into the study, to attend the training and supervision if allocated to the ERP arm of the trial, and to provide quantitative and qualitative feedback on the training and supervision.

Service users with a clinical diagnosis of Bipolar Disorder are initially informed about the study by their CC. We obtain lists of service users who would seem to meet the inclusion/exclusion criteria for the study according to the CMHT prior to the randomisation of teams to prevent any bias in the numbers of service users who are recruited to the two arms of the study. Recruitment occurring post-randomisation of the CMHTs is noted and rates of recruitment monitored to identify any post-randomisation selection bias or impact on recruitment rates. Service users are asked to consent to their contact details being passed to the research team in order to receive more information about the study. Those who consent are sent a letter and a visit is arranged with a researcher who gives verbal and written information about the study. Service users are then asked to give written consent in two parts. The first part consents them to take part in the data collection for the study, and the second to take part in the clinical intervention, if they are allocated to the ERP arm of the trial. This ensures that participants who drop out of the intervention, can still take part in the research study if they wish [16].

**Figure 1:**
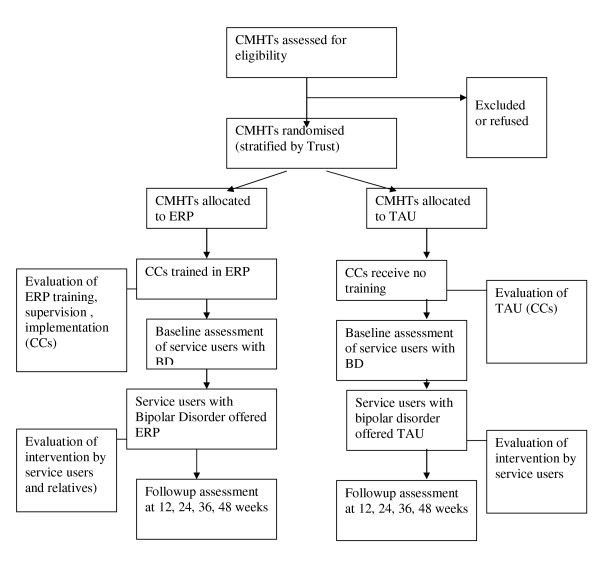
Diagram showing Design of the Study.

Service users offered ERP are invited to involve a relative/friend in the intervention. Where this occurs, the relative/friend is asked by the CC to give written consent to take part in the study. Following the intervention, the relative/friend is invited to give qualitative feedback on their experiences. Figure 1 gives an outline of the design of the study.

### The intervention

#### Enhanced relapse prevention

The intervention was initially devised by one of the authors (RM) [17] and has been developed further for this study by the PI (FL) one of the grant holders (PK) and the trial therapist (LT). A detailed manual outlining the rationale and process of the intervention is given to all CCs and service users. This forms the basis of a collaborative intervention.

#### Training and supervision

The training takes place over six 2-hour long sessions, occurring approximately weekly. The training is offered in NHS accommodation at a time and location most convenient to the teams being trained. The training follows the six-session format of the intervention and the aim of each session is to ensure the CCs understand the rationale for the content of each session and feel able to work with service users to achieve the session aims. The training includes a mixture of didactic information-giving, group discussion, videoed role-play examples (using professional actors), and in-situ role play tasks.

Following the training, all CCs are offered weekly clinical supervision for three months during which time they are expected to offer the intervention to at least one service user. The supervision is offered in groups consisting of CCs from the same team. All of the training and supervision sessions are conducted by the trial therapist (LS). CCs are asked to give feedback on the training and supervision. At the end of the intervention with the first service user, they are asked to rate their perceived competency and confidence in offering the intervention. Each individual is also rated by the trial therapist at the same time point.

### Data collection and analysis

A breakdown of reasons for exclusion and reasons for withdrawal from the training, intervention and follow-up phases of the study will be provided (Figure 1). Table 1 shows the timeframe for the assessments and measures that are used with service users with Bipolar Disorder. All measures are completed at face to face interview expect where indicated otherwise. Interviews take place in the service users' home, or a suitable NHS site.

**Table 1:** Schedule of Quantitative Assessments for Service Users

**Assessment**	**0 weeks (baseline)**	**12 weeks**	**24 weeks**	**36 weeks (by telephone)**	**48 weeks**
***Primary Outcome measures***					

Hamilton Rating Scale for Depression	+	+	+	+	+
Bech-Rafaelsen Mania Scale (MAS)	+	+	+	+	+
Current mental state coding (SCID-I)	+	-	-	-	-

***Secondary Outcome measures***					

LIFE scores for mania and depression for current week	+	+	+	+	+
Social and Occupational Functioning Assessment Scale (SOFAS)	+	+	+	-	+
Client Service Receipt Inventory (CSRI)	+	-	+	-	+

***Process measures***					

# Early Warning Signs Checklist (Mania and Depression)	+	+	+	-	+
# Coping strategies Checklist (Mania and Depression)	+	+	+	-	+
# Brief Illness Perception Questionnaire for Bipolar Disorder (modified IPQ)	+	+	+	-	+

***Potential confounds***					

Demographics	+	-	-	-	-
Clinical data*	+	+	+	-	+
Lifetime coding of mental illness (SCID-I)	+	-	-	-	-
SCID-II coding for Borderline and Antisocial Personality Assessment	+	-	-	-	-
Verona Service Satisfaction Scale (VSSS-EU)	+	-	-	-	+
Relationship Quality Rating	+	+	+	-	+

*Clinical data includes current medication, start and end dates of last episode and history of episodes over the previous 3 years.

# designed or significantly modified for this study

### Primary outcomes

The primary aims of the study are to assess the acceptability and feasibility of the trial design, and to estimate the effect size of the impact of the intervention on outcome.

#### Acceptability and feasibility

Recruitment, retention and attendance rates for CCs, service users, and relatives will be reported. Likert scale ratings of the quality of training and supervision and of CC competence and confidence in using ERP will be analysed. Barriers to carrying out the study at each stage will be identified and discussed and potential solutions identified.

Semi-structured in-depth interviews are conducted with a subsample of Care Coordinators and service users from each arm of the trial, and relatives involved in ERP. The aims are to: explore experiences of care coordinators of training and implementation of ERP within an NHS setting; to identify factors involved in the implementation and effectiveness of the ERP intervention for CC (at an individual, team and organisational level), service users and relatives or friends; to ascertain what TAU is for those with Bipolar disorder as received/provided by their care coordinators; and to explore the experiences of relatives/friends of service provision and their role in the management of Bipolar disorder.

A purposive maximum variation sampling strategy is deployed for each interview group and is an iterative process. Thus, on the basis of initial analysis, additional participants are selected who are thought to be able to fill-in, expand, or challenge previous data. At most 20 to 30 interviews will be conducted for each group although this may vary if data saturation is obtained, or if vagaries of data collection impede the attainment of theoretical saturation. Data are analysed using a grounded theory [18] approach. Following a constant comparative approach, an initial coding frame is complied, which is refined and elaborated in the light of incoming data and ongoing analysis. The results of the qualitative analysis will be reported elsewhere on completion of the trial.

#### Impact of the intervention on outcome

An estimate of the effect size of the intervention will be made from a comparison of time from baseline to recurrence of an episode of major depression, hypomania, mania, or mixed, satisfying DSM-IV criteria [19] following at least 8 weeks below this level for that pole. The number and percentage of patients satisfying criteria for a DSM-IV episode, broken down by type (major depression; mania type; other), will be reported for each treatment group and overall. The number and percentage of patients for whom information is imputed will also be reported. The intervals (in days) from baseline to recurrence (of any type) will be summarised by Kaplan-Meier curves (plotted up to 48 weeks). Cox proportional hazard models with robust variance estimators will be used to provide estimates of the hazard ratios and 95% confidence intervals.

The main analyses will be conducted on all patients from all teams (assigned to treatment groups (ERP and TAU) as randomised), applying the principle of intention to treat (ITT), as far as is practically possible, given any missing data. Sensitivity analyses based on a "per protocol" (PP) analysis will be conducted to examine robustness of the main results to departure from intended trial treatment. The sample of patients for the PP analyses is defined as the sample for the ITT analyses minus those patients who could not have received sufficient trial treatment due to CCs not attending at least 4 training sessions, or service users receiving less than 4 sessions of ERP. Given that the study is a feasibility study used in part to estimate the sample size for a larger study, effect sizes with 95% confidence intervals will be given weight rather than reliance on levels of statistical significance alone.

### Secondary outcomes

The effect of the intervention and the clustering design on secondary outcome and process measures will be assessed over 12 months. Secondary outcomes include symptom severity, social and occupational functioning, and cost of services received in each arm of the trial. Specific hypotheses linked to these are that (1) ERP will improve outcome compared to TAU by reducing the severity of symptoms; (2) ERP will improve outcome compared to TAU by improving social and occupational functioning; (3) there will be no difference in the overall cost of services received between those receiving ERP and those receiving TAU. Whilst it is recognised that ERP may initially require additional contact with the CC, following the intervention, service users who have received ERP will be better able to manage their own mood changes and are less likely to experience a relapse or require an admission to hospital.

Severity ratings of overall symptom levels based on the LIFE-II (Longitudinal Interval Follow-up Evaluation) will be obtained at weekly intervals post-baseline to 48 weeks. These will be averaged over intervals of four weeks, separately for depression and mania, and analysed by multilevel modelling to allow for the repeated measures and cluster design. Multilevel models will also be used to compare social and occupational functioning and cost of services, between groups.

### Process measures

Measures will be used to try to understand the process by which any changes occur. Process measures include recognition of early warning signs of relapse, use of coping strategies, and beliefs about mental health problems. Specific hypotheses linked to these measures are: (1) ERP will improve outcome compared to TAU by increasing the frequency of monitoring for early warning signs, and the number of signs identified in the early phase of relapse. (2) ERP will improve outcome compared to TAU by increasing the number and perceived effectiveness of appropriate coping strategies for managing early warning signs of relapse (Coping Strategy Checklist (modified from Lam & Wong 1997 [20]); (2) ERP will have a positive impact on service users beliefs about their mental health problems (Brief Illness Perception Questionnaire for Bipolar Disorder (modified from the IPQ Weinman et al 1996 [21]). Multilevel models allowing for repeated measures and cluster design will be used to compare monitoring and recognition of early warning signs, use of coping strategies and beliefs about mental health problems between the groups.

### Potential confounds

A number of potential confounds have been identified on the basis of previous work in this area including number of previous episodes [22,23], amount of social support [24] and use of medication [25]. These are assessed and will be controlled for in the analyses.

## Discussion

This study is essential in providing information necessary to the planning and execution of a definitive evaluation of the clinical effectiveness of relapse prevention approaches for Bipolar Disorder in the NHS.

We have devised an enhanced form of a relapse prevention approach that has already been shown to reduce relapse rates for mania. The enhancements are designed to: improve the effectiveness of the intervention at reducing rates of relapse into depression; make the intervention more widely available by tailoring it specifically to be used by CCs currently working in the NHS (rather than requiring referral to specialist services); streamline the intervention by identifying the key elements that can be offered in six, one-hour sessions; involve a friend or relative which is likely to increase the effectiveness of the intervention and make it more acceptable to service users. This intervention is highly consistent with the psychosocial components of treatment recommended by the recent NICE Clinical Guidelines for Bipolar Disorder [9].

We have designed the feasibility study to provide as much detailed and accurate information as possible. The inclusion criteria for staff and for service users are as wide as possible to increase generalisability. The manual and all training materials are designed to a high standard and quality for wider dissemination. Detailed feedback is sought on the training and supervision. The service user assessments include well standardised, valid and reliable measures of the key outcome variables, along with extensive qualitative interviews to explore the experiences of participants in depth. The research team meet on a regular basis to discuss and log barriers faced in conducting the research and ideas for how these could be avoided/overcome in future work. A detailed picture is obtained about how the intervention is used in practice.

There are a number of important limitations to the study that need to be addressed in future work. Firstly, this study is designed to assess the impact of clustering at the level of CMHT and to account for this in the statistical analysis of outcome. However, in reality the study has 2 levels of clustering: the CMHT and the Care Coordinator. Each Care Coordinator is asked to offer the ERP intervention to a minimum of 3 service users. It could be argued that data from service users who receive the intervention from the same Care Coordinator is likely to be correlated more highly than that from different Care Coordinators within the same team. However, due to the fact that the number of people within each cluster at the CC level would be likely to be very small i.e. 3, and the total sample would be insufficient to explore multiple level clustering, this will not be accounted for in the analysis.

Secondly, we have argued that a cluster design is required because ERP is essentially a team approach. Service users may need to be able to contact the CMHT at very short notice in response to increased recognition of important changes in their early signs of relapse. An effective and reliable response from the team is essential. For this reason, whichever CMHT staff member is currently on duty should be able to work with the ERP plan. In reality, we have recruited teams with a minimum of 4 CCs consenting to take part. This is for purely pragmatic reasons. In setting up the study, services felt unable to commit the time and resources for all staff to attend. Although the documentation regarding early signs monitoring and action plans is available in the service users notes, future work needs to address the best ways to disseminate information within and between mental health teams. It is possible that this limitation will reduce the effectiveness of the intervention.

Thirdly, due to the time constraints on the study, we are unable to recruit all service users prior to randomisation at each NHS Trust site. CCs make referrals throughout the study and generally offer ERP sequentially to one person at a time. Teams in TAU also make referrals throughout as more people who are eligible are identified. We are assessing and will report on whether or not this has led to a recruitment bias.

Fourthly, we are not able to assess fidelity to the intervention for all service users. The trial therapist makes a rating of adherence to the manual for the service users who are offered the intervention during the period of supervision. However, after completing the first intervention, CCs are offering ERP to service users outside of supervision. There is no assessment of fidelity for these interventions.

These limitations are clearly important and need to be addressed in future work. However, despite these, the results from this study are essential in progressing research and clinical innovation in this area. If we are able to show that it is feasible to train CCs in the NHS to offer ERP for people with Bipolar Disorder, it will be possible to design a large scale definitive trial to evaluate the effectiveness of the intervention on relapse rates. Such an intervention has the potential to offer service users a way of increasing control over their mood disorder and ultimately improving their quality of life and that of their relatives and friends.

## Competing interests

The author(s) declare they have no competing interests.

## Authors' contributions

FL was involved in the design, oversaw the implementation, produced initial draft of manual, and drafted the manuscript. CG provided statistical expertise in design, data collection and the manuscript. PK contributed to the design and supervision of data collection. LT developed the manual and a training package, provided the clinical training and supervision and collected data linked to this. CC managed and carried out recruitment and data collection. ET carried out recruitment and data collection and contributed to a draft of the manuscript. SP and EP jointly designed and managed the qualitative arm of the study. WS contributed to the training package and supervision of data collection. RM was involved in the design of the study, oversaw implementation of recruitment and clinical assessments, designed the training materials, and contributed to drafting the manuscript. All authors read and approved the final manuscript.

## Pre-publication history

The pre-publication history for this paper can be accessed here:



## References

[B1] Gitlin MJ, Swendsen J, Heller TI, Hammen C (1995). Relapse and impairment in bipolar disorder. American Journal of Psychiatry, 152, 1635-40.

[B2] Tohen M, Waternaux GM, Tsuang MT (1990). Outcome in mania: a 4-year prospective follow-up of 75 patients utilising survival analysis. Archives of General Psychiatry,.

[B3] Prien RF, Potter WZ (1990). NIMH workshop report on treatment of bipolar disorders.. Psychopharmacology Bulletins.

[B4] Lish JD, Dime-Meenan S, Whybrow PC, Price RA, Hirschfeld RMA (1994). The National MDA Survey of bipolar members. Journal of Affective Disorders,.

[B5] Hill RG, Shepard G, . PH (1996). A Survey of the Manic Depression Fellowship.

[B6] Morriss RK, Faizal MA, Jones AP, Williamson PR, Bolton C, McCarthy JP Interventions for helping people recognise early signs of recurrence in bipolar disorder.. Cochrane review.

[B7] Bauer MS, McBride L, Williford WO, Glick H, Kinosian B, Altshuler L, Beresford T, Kilbourne AM, Sajatovic M (2006). Collaborative care for bipolar disorder: Part II. Impact on clinical outcome, function, and costs. Psychiatr Serv.

[B8] Bauer MS, McBride L, Williford WO, Glick H, Kinosian B, Altshuler L, Beresford T, Kilbourne AM, Sajatovic M (2006). Collaborative care for bipolar disorder: part I. Intervention and implementation in a randomized effectiveness trial. Psychiatr Serv.

[B9] National Institute for Health and Clinical Excellence (2006). Bipolar disorder: the management of bipolar disorder in adults, children and adolescents, in primary and secondary care. NICE clinical guideline 38.. NICE Guidelines.

[B10] Perry A, Tarrier N, Morriss R, McCarthy E, Limb K (1998). Randomised controlled trial of efficacy of teaching patients with bipolar disorder to identify early symptoms of relapse and obtain treatment. British Medical Journal.

[B11] Department of Health Mental Health Policy Implementation Guide. Community Mental Health Teams.. http://www.doh.gov.uk/mentalhealth.

[B12] Thornicroft G, Wykes T, Holloway F, Johnson S, Szmuckler G (1998). From efficacy to effectiveness in community mental health services. PriSM Psychosis study.. British Journal of Psychiatry,.

[B13] Medical Research Council (2000). A framework for development and evaluation of RCTs for complex interventions to improve health.. MRC publications.

[B14] Eldridge SM, Ashby D, Feder GS, Rudnicka AR, Ukoumunne OC (2004). Lessons for cluster randomised trials in the twenty-first century: a systematic review of trials in primary care. Clinical Trials.

[B15] Baldwin SA, Murray DM, Shadish WR (2005). Empirically supported treatments or type I errors? Problems with the analysis of data from group-administered treatments. J Consult Clin Psychol.

[B16] Hutton JL (2001). Are distinctive ethical principles required for cluster randomised controlled trials?. Statistics in Medicine.

[B17] Morriss R (2004). The early warning symptom intervention for clients with bipolar affective disorder. Advances in Psychiatric Treatment,.

[B18] Glaser B, Strauss A (1967). The discovery of grounded theory..

[B19] American Psychiatric Association (1994). Diagnostic and Statistical Manual of Mental Disorders.

[B20] Lam DH, Wong G (1997). Prodromes, coping strategies, insight and social functioning in bipolar affective disorders. Psychological Medicine.

[B21] Weinman J, Petrie K, Moss-Morris R, Horne R (1996). The Illness Perception
Questionnaire: a new method for assessing the cognitive representation of illness.. Psychology and Health.

[B22] Scott J, Paykel E, Morriss R, Bentall R, Kinderman P, Johnson T, Abbott R, Hayhurst H (2006). Cognitive-behavioural therapy for bipolar disorder. Br J Psychiatry.

[B23] Scott J, Paykel E, Morriss R, Bentall R, Kinderman P, Johnson T, Abbott R, Hayhurst H (2006). Cognitive-behavioural therapy for severe and recurrent bipolar disorders.. British Journal of Psychiatry.

[B24] Miklowitz DJ, George EL, Richards JA, Simoneau TL, Suddath RL (2003). A randomised study of family-focused psychoeducation and pharmacotherapy in the outpatient management of bipolar disorder. Archives of General Psychiatry,.

[B25] Colom F, Vieta E, Sanchez-Moreno J, Martinez-Aran A, Reinares M, Goikolea JM, Scott J (2005). Stabilizing the stabilizer: group psychoeducation enhances the stability of serum lithium levels. Bipolar Disord.

